# Multifunctional Dyeing of Wool Fabrics Using Selenium Nanoparticles

**DOI:** 10.3390/polym14010191

**Published:** 2022-01-04

**Authors:** Tarek Abou Elmaaty, Sally Raouf, Khaled Sayed-Ahmed, Maria Rosaria Plutino

**Affiliations:** 1 Department of Material Arts, Faculty of Art & Design, Galala University, Galala 43713, Egypt; tasaid@gu.edu.eg; 2 Department of Textile Printing, Dyeing & Finishing, Faculty of Applied Art, Damietta University, Damietta 34511, Egypt; 3 Department of Textile Printing, Dyeing & Finishing, Faculty of Applied Art, Banha University, Banha 13518, Egypt; soola.a@hotmail.com; 4 Department of Agricultural Chemistry, Faculty of Agriculture, Damietta University, Damietta 34511, Egypt; dr_khaled@yahoo.com; 5 Consiglio Nazionale Delle Ricerche, c/o Dipartment ChiBioFarAm, Istituto per lo Studio dei Materiali Nanostrutturati, University of Messina, Viale F. D’Alcontres 31, Vill. S. Agata, 98166 Messina, Italy

**Keywords:** multifunctional finishing, selenium nanoparticles, wool fabrics, UV-blocking properties, antimicrobial activity

## Abstract

This work aims to utilize selenium nanoparticles (Se-NPs) as a novel dyestuff, which endows wool fibers with an orange color because of their localized surface plasmon resonance. The color characteristics of dyed fibers were evaluated and analyzed. The color depth of the dyed fabrics under study was increased with the increase in Se content and dyeing temperature. The colored wool fabrics were characterized using scanning electron microscopy (SEM), energy dispersive spectroscopy (EDX) and an X-ray diffraction (XRD) analysis. The results indicated that spherical Se-NPs with a spherical shape were consistently deposited onto the surface of wool fibers with good distribution. In addition, the influence of high temperature on the color characteristics and imparted functionalities of the dyed fabrics were also investigated. The obtained results showed that the proposed dyeing process is highly durable to washing after 10 cycles of washes, and the acquired functionalities, mainly antimicrobial activity and UV-blocking properties, were only marginally affected, maintaining an excellent fastness property.

## 1. Introduction

Recently, the usage of unique dyes and finishing agents has had a substantial influence on textiles’ functionality. The dyeing of textiles with multi-functional characteristics has attracted significant interest in recent years. Nanotechnologies and nanomaterials offer a wider application potential for preparing functional textiles, such as flame retardant, self-cleaning, wrinkle-resistant, antistatic, antimicrobial, and UV-protective, etc. [1,2,3,4,5,6,7,8,9,10,11,12,13,14] In recent years, several approaches to metal and metal oxide NP fabrication and application have been developed to impart functionalities to different types of textile substrates [15,16,17,18,19,20,21,22,23]. In addition, nanomaterials, such as gold and silver nanoparticles, stabilized by opportune functional capping agents, have been used as a novel class of functionalizing nanofillers either themselves [24,25,26,27,28,29] or for the functionalization of different types of materials (such as concrete, glass, geopolymer and also embedded in coloring or antibacterial coatings), especially for textile fabrics [30].

The coloration of fabric/fiber can be achieved through a combination with metal nanoparticles by means of the particular optical properties of plasmon nanomaterials, called localized surface plasmon resonance (LSPR), producing brilliant and vivid colors, which are different from traditional dyes in that they are not the chromophore of traditional dyes but the shape and size of nanoparticles that determine the colors [31,32]. This has recently motivated research activities to directly employ metal nanoparticles in the dyeing of different textile substrates to overcome color fading and high water and energy consumption problems associated with conventional dyeing methods. Furthermore, excessive exposure to dyes may cause respiratory problems and skin irritation and increase risk of cancer [33]. Hence, several different approaches have been developed to impart colors and functionalities to diverse fabrics/fibers, such as cotton, wool [34,35,36,37,38,39,40,41], silk [42,43,44,45], bamboo [46], ramie [47], viscose and acrylic [48] using noble metal nanoparticles via a self-assembly or in-situ synthesis methods.

Owing to the localized Surface Plasmon Resonance (LSPR) property of selenium nanoparticles (Se-NPs), they exhibit adaptable colors depending on the synthesizing measures. Besides, Se-NPs have strong cytotoxicity towards a broad range of microorganisms, low toxicity to human cells, high selectivity, and long-term durability [49]. Se-NPs have significant antimicrobial and antioxidant activity, which have been widely reported in the literature [50,51,52], and their applications in textile fabrics have been reported once by Joanne Yip et al. [53] as an antimicrobial agent for polyester fabric.

In our previous study, cotton and polyester were colored with silver and gold NPs via a printing technique to render different functions of fibrous materials [54]. Subsequently, Se-NPs were used in the wool fabric printing process to fabricate antimicrobial textiles with low cytotoxicity [55]. In the current study, wool fabrics were dyed with Se-NPs in a different way to produce multifunctional fabrics with brilliant stable colors in one step and compare between the results obtained from both dyeing and printing techniques.

## 2. Experimental

### 2.1. Materials

The fabrics used here were 100% scoured and bleached wool. Sodium hydrogen selenite, vitamin C and polyvinylpyrrolidone (PVP) were purchased from *Loba Chemie, India*. In addition, the other chemicals used in this study were of commercial grade.

### 2.2. Dyeing Apparatus

Dyeing was carried out using an Infra Color Dyeing Machine, which consists of 12 beakers that are mounted in a rotating beaker-carrying wheel. Heating occurs through infrared radiation, cooling through air and automation through the microprocessor programmer DC4 F/R. The maximum temperature was up to 140 °C, a maximum heating rate was up to 5 °C/min and cooling had a maximum rate up to 3 °C/min.

### 2.3. Methods

#### 2.3.1. Green Synthesis of Se-NPs

Se-NPs were prepared according to the method reported by Abou Elmaaty et al. [55] through a redox reaction. Sodium hydrogen selenite at different concentrations of 25 mM, 50 mM, 75 mM and 100 mM was added to vitamin C at the same concentration and the volume ratio of 1:1 under magnetic stirring. PVP was used as a stabilizer at the concentration of 0.75–3 g/100mL of vitamin C to enhance the stability of Se-NPs. The change in color from colorless to orange to dark orange indicated the formation of Se-NPs.

#### 2.3.2. Dyeing Procedure of Wool Fabrics with Selenium NPs

Wool fabrics were dyed with different concentrations of Se-NP solution using a wet chemistry method through an immersion process. Briefly, the wool fabrics were immersed into the Se-NP solutions (dyeing solutions) without any chemical additives. Dyeing was performed in a laboratory-scale thermal HT dyeing machine with a liquor-to-goods ratio of 100:1. The solutions containing Se-NPs and wool fabrics were shaken at different temperatures, including room temperature, 40 °C, 70 °C and 100 °C for 60 min to obtain the coloration of fabrics. At the end of the dyeing process, the dyed wool samples were removed, rinsed with water and dried at room temperature.

### 2.4. Characterization

TEM images of the synthesized Se-NPs were obtained using a JEM-2100 Transmission Electron Microscope (TEM) with an acceleration voltage of 200 kV to determine the size and morphology of the NPs. Samples for the TEM analysis were prepared by dripping a drop of Se-NP solution onto a carbon-coated copper grid and drying at room temperature.

The surface morphology of wool fabrics, either blank or dyed fabrics, was examined using high resolution scanning electron microscopy (JEOL JSM-6510LB with a field emission gun, Tokyo, Japan). The chemical structure of the dyed samples was analyzed using a surface energy dispersive spectroscopy (EDX) analysis unit (EDAX AMETEK analyzer, Rigaku, Japan) attached to the electron microscope.

X-ray diffraction was analyzed for both the prepared Se-NPs and Se-dyed wool fabrics using an X-ray diffractometer system (XRD) (Bruker D8 ADVANCE, Karlsruhe, Germany). The Se-NP solution was dried at 130 °C until completely dried.

### 2.5. Functional Properties of Se-Dyed Wool Fabrics

The functionalities of wool fabrics dyed with Se-NPs were evaluated in terms of the UV protection factor (UPF) and antimicrobial activity.

#### 2.5.1. Antimicrobial Activity

The biological activity of the Se-dyed wool samples was evaluated qualitatively against G+ve bacteria (Staphylococcus aureus and Bacillus cereus), G-ve bacteria (Escherichia coli) and yeast (Candida utilis). The antimicrobial test was performed according to the AATCC Test Method (147–1988). The antimicrobial activity was expressed as the growth inhibition zone (mm).

#### 2.5.2. UV-Protection Properties

The ultraviolet protection factor expressed as UPF and transmission of ultraviolet (UV) radiation through wool fabrics were evaluated based on the AS/NZS 4399:1996 test method. The UV protection was rated as good, very good or excellent if the UPF values were between 15–24, 25–39 or above 40, respectively.

### 2.6. Testing

#### 2.6.1. Color Measurements

The color coordinates of CIE lab (L *, a *, b *, C *, and h*) and color strength (K/S) for both blank and Se-dyed wool fabrics were measured in the wavelength range of 360–720 nm using a Konica Minolta spectrophotometer (CM-3600 d, Minolta, Tokyo, Japan). All samples were measured in three different areas, bearing in mind both sides of the fabrics, and the mean values were recorded.

#### 2.6.2. Fastness Properties

The color fastness properties were evaluated for all Se-dyed wool fabrics in accordance with the standard test method. Washing, rubbing and light fastness were measured according to the method of AATCC test methods (61–1972), (8–1972) and (16A–1972), respectively.

#### 2.6.3. Durability Test

Durability to washing of the imparted functionalities (antimicrobial activity and UV-protection properties) as well as color strength (K/S) of Se-dyed wool fabrics were evaluated according to AATCC Test Method 61(2A)-1996 after 10 laundering cycles.

#### 2.6.4. Mechanical Properties

The tensile strength (maximum load) and elongation at break (maximum strain) of blank and Se-dyed wool fabrics at high temperature were evaluated using the strip test method on a multi-tester machine according to ASTM-D4850 with a load cell 500 N, preload 0.01 N, speed of 100 mm/min and gauge length of 100 mm.

### 2.7. Statistical Analysis

Antimicrobial, UV-protection, and mechanical tests were performed by taking the average of three readings (samples). The standard error of the mean was calculated according to the equation given below and was found to be + (−) 0.1 SEX = S/√n

Where S = sample standard deviation and n = the number of observations of the samples.

## 3. Results and Discussion

The dyeing process of wool fabrics using selenium nanoparticles (Se-NPs) as a new functional colorant was investigated to obtain multifunctional dyed fabrics based on colloidal solutions of nanomaterials. The influences of different dyeing temperatures on the adsorption of Se-NPs onto wool fabrics and alteration of the color characteristics of wool samples were observed. The imparted functionalities were also determined. The results obtained and appropriate discussions are presented below.

### 3.1. Characterization of Se-NPs and Dyed Wool Fabrics

#### 3.1.1. Transmission Electron Microscopy (TEM)

**Figure 1:**
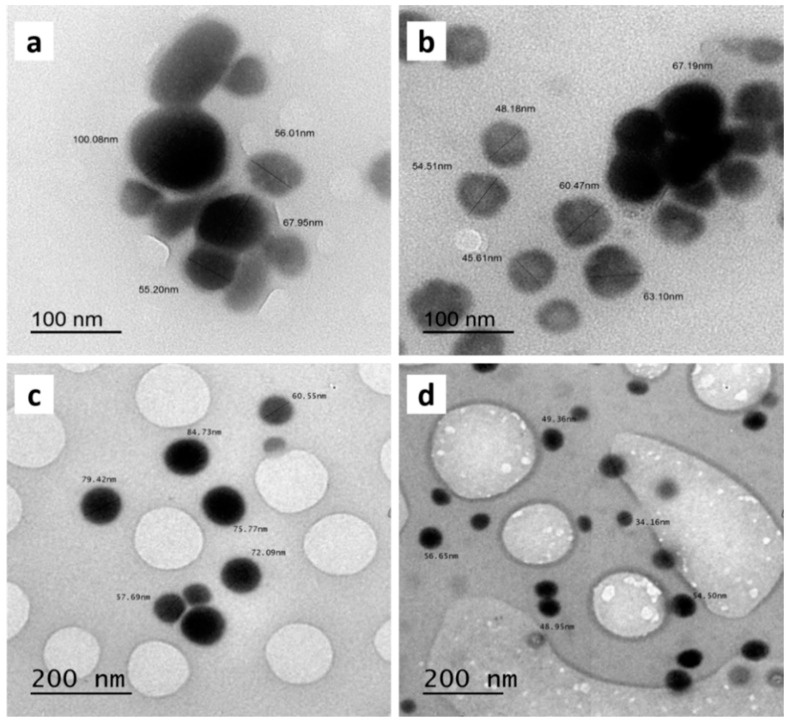
TEM images of synthesized Se-NPs on different concentrations: (**a**) 12.5 mM, (**b**) 25 mM, (**c**) 37.5 mM and (**d**) 50 mM.

Figure 1 shows TEM images of colloidal Se-NPs prepared at different concentrations. The obtained TEM micrographs revealed that the Se-NPs prepared at a concentration of 50 mM had the lowest diameter range (25–90 nm) compared to the other prepared Se-NPs.

The Se-NPs were well-dispersed and mostly spherical in shape when no agglomeration or deformation of Se-NPs were observed. The Se-NPs prepared at a concentration of 12.5 mM showed the largest size up to 115 nm, resulting in the appearance of a block structure, as shown in Figure 1a. While the concentration of the stabilizer PVP (3 g/100mL) and sodium hydrogen selenite increased, the shape of the Se-NPs was observed to be uniformly spherical instead of an aggregated form and had the lowest diameter, as shown in Figure 1d.

#### 3.1.2. SEM and EDX Analysis

**Figure 2:**
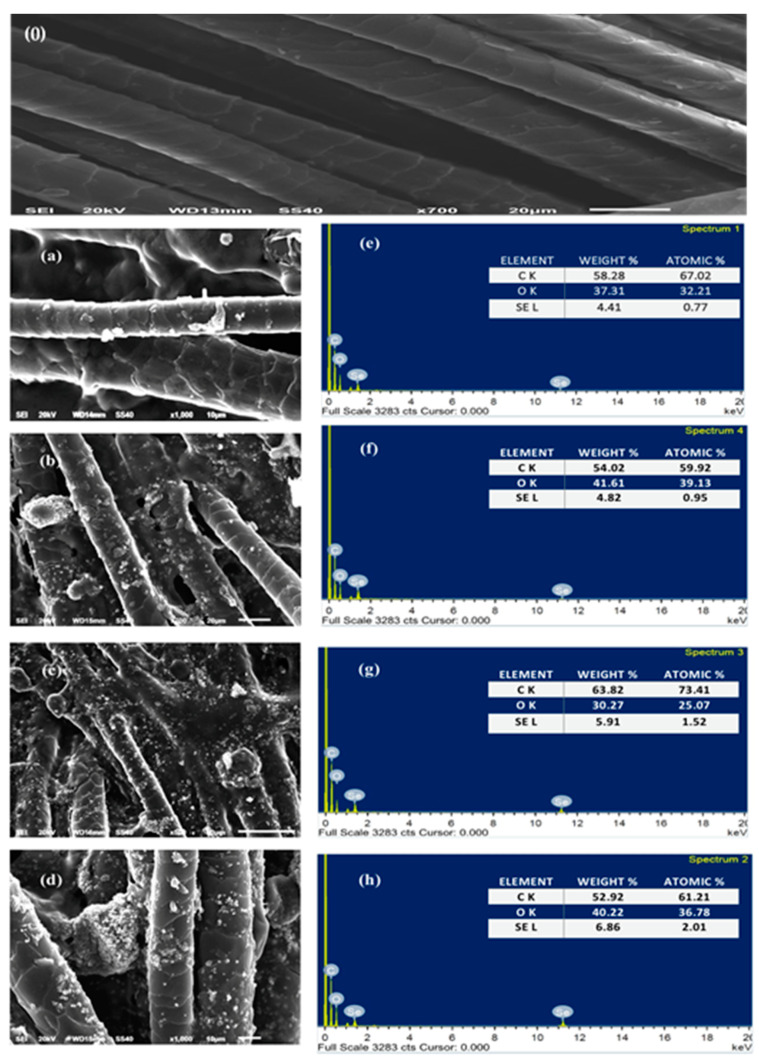
SEM micrographs of (**0**) blank wool fabric, and (**a**–**d**) wool fabrics dyed with Se-NPs at different concentrations of: (**a**) 12.5 mM, (**b**) 25 mM, (**c**) 37.5 mM and (**d**) 50 mM, as well as EDX spectra of Se-dyed wool fabrics with varied concentrations of: (**e**) 12.5 mM, (**f**) 25 mM, (**g**) 37.5 mM and (**h**) 50 mM.

In Figure 2, SEM images show the topographical characteristics of the blank and Se-dyed wool fabrics. The SEM images of the blank wool show typically clear, clean scales and a smooth longitudinal fibrous structure surface, as shown in Figure 2(0). The SEM images of the surface of the Se-dyed wool fabrics revealed that the surfaces of wool fabrics were covered by a sufficient layer of Se in the nano size, and Se-NPs on the wool fabric surfaces had a wide range of size distribution.

The surface chemical elements of the Se-dyed wool fabrics were determined by EDX spectroscopy, as shown in Figure 2e–h. The peaks appearing at about 1 and 11 Kev in each figure are attributed to selenium NPs. The SEM images and EDX results provide strong evidence for the formation of Se-NPs on the surface of the wool fabrics.

#### 3.1.3. X-ray Diffraction (XRD)

**Figure 3:**
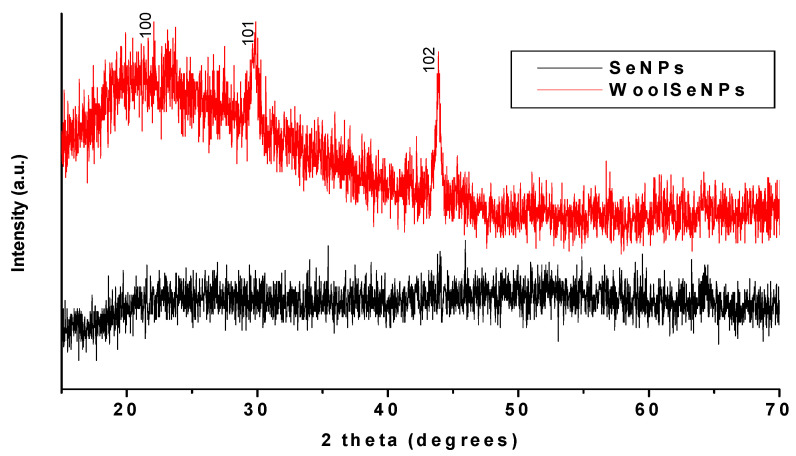
XRD patterns of the synthesized Se-NPs and Se-dyed wool fabric.

An XRD analysis was performed for further confirmation of the Se-NP formation. This analytical method aids in the determination of crystallite materials and provides details of the unit cell dimensions. As shown in Figure 3, the obtained Se-NPs for both prepared solutions and Se-dyed wool fabrics were highly crystalline, and all diffraction peaks were well indexed as 24.28°, 29.24° and 43.64°, corresponding to 100, 101 and 102 crystal planes, respectively, in accordance with the JCPDS 86-2246 international database. [56] The results obtained from the SEM, EDX and XRD analyses confirmed the sufficient deposition of Se-NPs on the wool fabric surface.

### 3.2. Functional Properties of Se-NP Dyed Wool Fabrics

#### 3.2.1. Biological Activity

The large surface area of natural textile fabrics and their ability to retain moisture provide an excellent environment for microorganism growth. Therefore, imparting the antimicrobial properties for natural fabrics is of great interest. Thus, the antimicrobial activity of the unwashed and washed Se-dyed wool fabrics was evaluated qualitatively against G+ve (*S. aureus* and & *Bacillus cereus*), G-ve (*E. coli*) and yeast (*Candida utilis*). The results are reported in Table 1 and are expressed as the zone of growth inhibition ZI (mm).

From the results, it is quite clear that the blank wool fabric did not show any antimicrobial effect. However, the Se-dyed wool showed a notable antimicrobial activity that increased with the increase in Se-NP concentration against the tested pathogens. In this respect, SEM micrographs illustrated that the deposition of Se-NPs on the wool surface increased with the increase in Se-NP concentration, confirming the results obtained from the antimicrobial test. This enhancement may be due to the adsorption of Se-NPs, leading to cell wall depolarization, which changes the typically negative charge of the wall to become more permeable, and then, inhibition of cell membrane metabolisms causing the death of bacteria, and/or the increase in NP concentrations leading to a concomitant increase of highly reactive oxygen radicals causing the destruction of the molecular structure of bacteria [57].

On the other hand, the antimicrobial activity of the Se-dyed samples showed a slight decrease with an increase in the dyeing temperatures, which may be attributed to the significant impact of heat treatment on the size, structure and bioactivity of Se-NPs. Typically, smaller NPs have higher antimicrobial activity; this phenomenon can be explained by the small NPs having a larger surface area than larger NPs, which can extremely increase the production of highly reactive oxygen species (ROS), which severely damaged and inactivated the essential biomolecules, including proteins, DNA and lipids [57,58]

Moreover, subjecting the Se-dyed samples to up to 10 cycles of consecutive laundry cycles according to AATCC Test Method 61(2A)-1996 led to a non-sense decrement in their antimicrobial properties. Colored wool fabrics with low amounts of Se-NPs were enough to achieve excellent antimicrobial activity.

On the other hand, Adomaviciute et al. (2016) revealed that no antimicrobial activity was observed for the PVP solution (8%) against *Staphylococcus aureus*, *Bacillus cereus*, *Escherichia coli* and *Candida albicans* [59]. However, the PVP concentration required for Se-NPs preparation did not exceed 1.5% in this study, indicating that the antimicrobial activity mainly corresponded to Se-NPs.

These results demonstrated the activity of Se-NPs for ingrain dyed wool fabrics against the microbial pathogens and confirmed the high efficiency of the current processing in the acquisition of wool fabrics’ bio-functionality, in addition to the ingrain coloration of fabrics.

#### 3.2.2. UV-Protection Properties

Skin diseases, including allergies, premature skin aging, sunburn, and skin cancer, can occur due to skin exposure to UV irradiation. A great number of approaches were done to protect human skin against the harmful effects of UV irradiation using nanoparticles [60,61,62]. Therefore, the UV-blocking properties of the wool fabrics dyed with Se-NPs were evaluated. Table 2 showd the transmittance values of UV light in the range of UV-A (315–400 nm) and UV-B (280–315 nm) ranges and the UV protection factor (UPF) values of the undyed wool fabric and the wool fabrics dyed with Se-NPs. The average transmittance values of the wool fabrics were decreased obviously after wool fabrics were dyed using Se-NPs, which confirmed that the Se-NPs prominently enhanced the UV-blocking activity of wool fabrics. Both transmittance values in the UV-A and UV-B regions decreased with increasing concentrations of Se-NPs on the wool fabrics.

**Figure 4:**
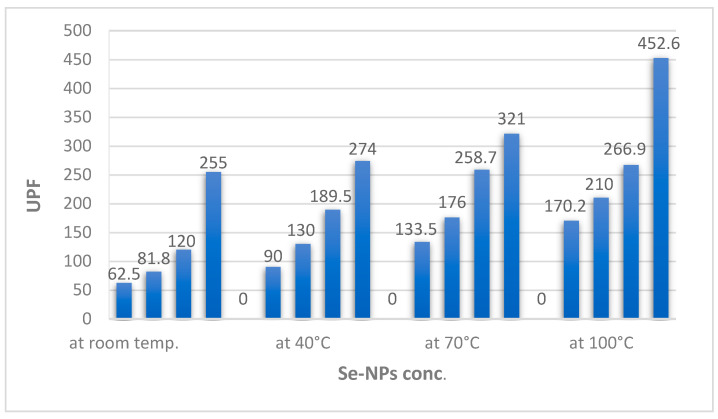
UV protection properties of Se-dyed wool fabrics at different temperatures.

The UPF refers to the fabric efficiency to block out UV irradiation from passing through and reaching the skin. The UPF value of the undyed wool fabrics was also measured to be 33.46 (Table 2). From the results obtained, it was obvious that the dyeing process with Se-NPs increased the UPF value of wool fabrics, as shown in Figure 4 and Table 2. The results confirmed that the Se-NP dyed wool fabrics provide excellent UV protection, which increased by increasing the Se concentrations.

Additionally, the influence of dyeing temperature on the UV protection properties of wool fabrics was examined. The UPF value of the wool fabrics were increased dramatically, as shown in Figure 4, as well as the UV light transmittance values were decreased with an increase in dyeing temperature. This may be due to the larger size of Se particles because of high temperature coloration; it could be considered that enlarged particles have a better chance to reflect more radiation [45].

Furthermore, the results in Table 2 also showed that repeated laundering for up to 10 cycles of the Se-dyed samples, evaluated according to AATCC Test Method 61(2A)-1996, caused a slight decrease in the imparted UV-protection functionality, expressed as UPF values; however, it was still rated as excellent values.

### 3.3. Color Characteristics and Color Fastness of Se-Dyed Wool Fabrics

**Figure 5:**
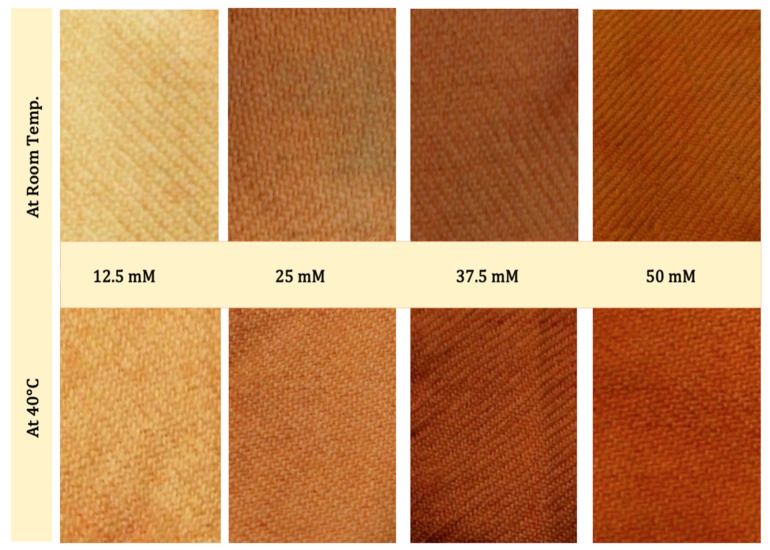
Photographs of Se-dyed wool fabrics with different concentrations of synthesized Se-NPs at room temperature and 40 °C.

**Figure 6:**
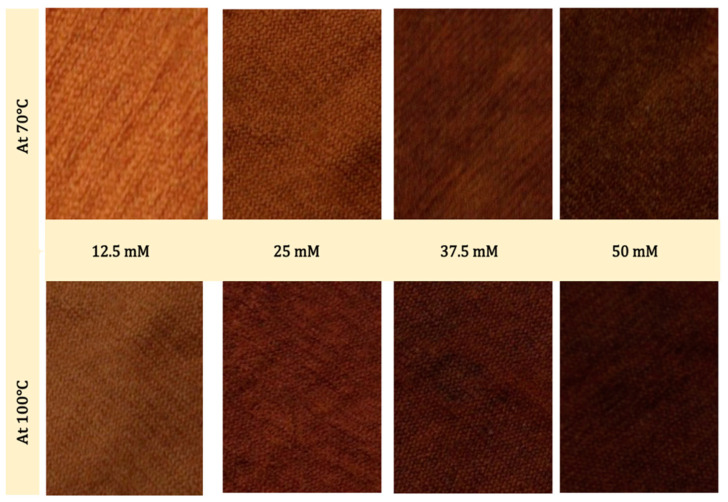
Photographs of Se-dyed wool fabrics with different concentrations of synthesized Se-NPs at 70 °C and 100 °C.

At the end of the dyeing process, the color of the wool fabrics was changed, and the fabric color reflected the color of the impregnated Se-NPs, as shown in Figure 5 and Figure 6. Color characteristics were quantified by estimating the color space in terms of L *, a *, b * and color strength (K/S). In this system, L * refers to lightness/darkness values from 100 to 0 representing white to black, a * values run from negative (green) to positive (red) and b * values run from negative (blue) to positive (yellow). The detected data are represented in Table 3. An illustration of the color data can be pointed in the following: dyeing at room temperature and 40 °C resulted in linear decrement in lightness values with increment in Se content, which was reflected in the color acquired by the incorporation of Se-NPs on wool fabrics. The lightness results were reduced from 79.77 to 59.14 at room temperature and from 75.56 to 42.32 for dyeing at 40 °C by increment in Se content from 12.5 to 50 mM.

Red/green values (a *) were found to be raised in a positive direction by the increment in Se content, which might have been attributed to acquiring the red color by ingrain clustering of Se-NPs, and the yellow/blue ratio (b *) was slightly increased by increasing the content of Se, reflecting the redness/yellowness color of the fabrics, as shown in Figure 5, which increased with Se content.

By increasing the dyeing temperature from 40 °C to 70 °C and 100 °C, the wool fabrics became red or brown, as indicated by the positive a* and b* values. The color of the wool fabrics darkened and changed to brown as the Se content and dyeing temperature increased, as shown in Figure 6, which indicated that temperature had a visible influence on the colors of Se-NP dyed wool fabrics, and this might be due to the increase on the sizes of Se-NPs when the solution was subjected to heat treatment [58]. The color strength (K/S) increased with the increase of dyeing temperature and Se-NP concentration. The maximum intensity of K/S was recorded in the wavelength range of 360–390 nm.

From the observations, it could be concluded that the dyeing process of wool fabric using synthesized Se-NPs resulted in the change of the fabric’s color from the original creamy-white color to a yellowish red (orange) color, varying to a brownish color, according to the increment in dyeing temperature and the concentration of Se-NPs. The significant increase in color depth with increasing Se content confirmed the main responsibility of Se-NPs in the ingrain coloration of wool fabrics due to the SPR effects of Se-NPs.

Moreover, Table 3 revealed that the color depth (K/S) of the Se-dyed samples were still high after 10 washing cycles of successive laundry cycles. This indicates that the Se-NPs were still tightly loaded and fixed onto the simultaneous functional dyeing of the wool fabric surface. This might be due to the electrostatic interaction between wool fibers, which protonated to carry positive charges and Se-NPs carrying negative charges, leading to the coloration of fibers.

The washing, rubbing and light color fastness of dyed wool fabrics with Se-NPs at different dyeing temperatures were evaluated according to AATCC standard test methods (61–1972), (8–1972) and (16A–1972), respectively.

The results represented in Table 4 showed washing, rubbing and light fastness properties for all Se-dyed samples. Both Se-dyed wool fabrics at room temperature and 40°C exhibited excellent results for washing and rubbing fastness, which agreed with the K/S data after the washing process, while the light fastness showed a very good rating of 4/5.

On the other hand, wool samples dyed at high temperatures (70 and 100 °C) exhibited lower color fastness properties. While washing fastness had a very good rating in both color alteration and staining, the rubbing fastness showed good results, and the wet one had a rate of 4 at 70 °C to 3/4 at 100 °C. In addition, the light fastness exhibited a very slight color fading rate of 4. It is well known that the larger the colorant (in size), the poorer the fastness for the colored fabrics. Therefore, the moderate fastness properties of dyed wool fabrics at a high temperature could be attributed to the enlarged size of Se-NPs onto the fabric surface, caused by heat treatment and, consequently, easy leaching [45,58]

Moreover, a washing durability test was conducted according to AATCC Test Method 61(2A)-1996, and the results indicated that the fastness properties of all Se-dyed wool fabrics were still high, even after 10 cycles of consecutive laundry cycles. These results demonstrated that the wool fabrics dyed with synthesized Se-NPs have very good color fastness properties.

### 3.4. Mechanical properties of Se-Dyed Wool Fabrics

Tensile strength (maximum load N/cm^2^) and elongation at break (maximum strain %) were both evaluated for blank and dyed wool fabrics to give an indication for the change in mechanical properties of wool fabrics after dyeing with Se-NPs at a high temperature (100 °C). The results are reported in Table 5. The tensile strength as well as the elongation at break slightly decreased after the dyeing process.

Tensile strength decreased from 356.8 N/cm^2^ for the blank wool to 176.6 N/cm^2^ and elongation at break for the blank sample also decreased from 26.95% to 13.88% for the applied concentration of 50 mM Se-NPs at 120 °C. This decrease in mechanical properties is probably related to high temperature dyeing processes, which could damage the chemical structure of the wool fiber, which means that 100 °C is the maximum temperature for dyeing wool fabrics with Se-NPs.

All experimental findings clearly show the great efficiency of the proposed dyeing process in terms of color fastness combined with improved functionalities, such as antimicrobial activity and UV-blocking properties. These results lay the groundwork for a durable dyeing of different kinds of fabrics by use of the simple impregnation with colloidal solutions of metallic nanoparticles prepared with a sustainable method. This represents an important result, considering that, today, the main worldwide pollution of white waters is produced by textile treatments and dyes. Furthermore, the pollution produced by the textile industry, employing a large consumption of water and the use of harmful dyes (often azo dyes), is harmful for the environment and for human health, with a huge negative impact on the planet. Often, wastewater is not adequately treated to remove pollutants before being discharged into the environment. Very recently, specific, and targeted treatments have been developed as a suitable method for the removal of azo dyes from textile effluents. Many recent studies refer to the use of photocatalytic degradation under visible light, the employment of non-living cells of marine microalgae (i.e., *Nannochloropsis oceanica*) and other waste materials, with a careful optimization of their response surface methodology, as a suitable and quite valuable wastewater treatment [63,64,65].

## 4. Conclusions

The present study has reported a one step process for the multi-functionalization of dyeing wool fabrics using a colloidal solution of Se-NPs. All Se-NP concentrations were adsorbed onto wool fabrics even though the dyeing temperatures were different, which was confirmed by the color fastness properties and color strength (K/S). The obtained colored wool fabrics had a stable bright color ranging from light orange to a dark orange color according to the concentration of the prepared Se-NPs as well as the dyeing temperatures. Moreover, the wool fabrics with Se-NPs exhibited significant antimicrobial activity and excellent UV protection properties with excellent washing durability up to 10 laundering cycles. XRD, SEM and EDX analyses revealed that the Se-NP solution was effectively absorbed on wool fabric surfaces. Furthermore, a high dyeing temperature led to a change in the color characteristics from a dark orange color to dark brownish color, especially at 100 °C. However, the UV-protection properties were not affected by high temperature dyeing; the antimicrobial properties slightly decreased, and the mechanical properties of the dyed wool fabrics also decreased when the dyeing temperature was increased. The study concluded that the Se-wool dyeing process at a low temperature (at room temperature or 40 °C) was better than that of high temperature dyeing. The proposed dyeing process of wool fabrics with Se-NPs demonstrated that the dyeing of fibers based on nanoparticles may facilitate the functionalization of fibrous materials better than the traditional dyestuffs that require energy and an increased temperature for dyeing. This work will open the way to the use of opportune nanoparticles for the functionalization of different kinds of surfaces, to obtain implemented mechanical properties (i.e., color, sensing and antibacterial).

## Figures and Tables

**Table 1:** Antimicrobial activity of the Se-dyed wool fabrics at different temperatures.

Se-NPs Conc.	Temp. °C	Antimicrobial Activity ZI ^1^ (mm.)			
Blank wool		G+ve		G−ve	Yeast
		*S. aureus*	*Bacillus cereus*	*E. coli*	*Candida utilis*
		0	0	0	0
12.5 mM	At room temp.	13.4 (13) ^2^	10.6 (9)	10 (10)	14 (13.5)
25 mM		15.9 (15)	12.6 (12)	13.5 (12.4)	17.5 (16)
37.5 mM		17.7 (17)	14 (13.2)	14.3 (14)	19 (18)
50 mM		19 (18)	15.4 (14.8)	18.2 (18)	23 (22.3)
12.5 mM	At 40 °C	13 (13)	10.2 (10)	9.3 (9)	14 (12)
25 mM		14.7 (13.8)	12 (11)	12.5 (11)	16.6 (15)
37.5 mM		17 (15)	13.8 (13)	14 (12.3)	18.5 (15.3)
50 mM		18.2 (17.4)	15 (14.3)	17.4 (15.6)	20.8 (20)
12.5 mM	At 70 °C	10 (8)	8 (8)	8.6 (7.2)	12 (10)
25 mM		11.5 (11)	10 (8.5)	9.7 (9)	12.8 (11.2)
37.5 mM		13 (10.3)	10.5 (10)	10.5 (9.2)	14 (12.6)
50 mM		14.8 (14)	12 (10)	11.5 (11)	17.2 (15.4)
12.5 mM	At 100 °C	8 (7)	7.6 (6.3)	7 (6)	10.6 (10)
25 mM		9 (8.4)	8.2 (7)	8.5 (7)	11 (9.3)
37.5 mM		11 (10.5)	8.8 (8)	9 (8.2)	11.8 (11)
50 mM		11.8 (10)	10 (8.8)	10 (9)	13.5 (12.6)

^1^ ZI (zone of inhibitions). ^2^ Values in parentheses indicate durability of antimicrobial activity after 10 washing cycles.

**Table 2:** UV protection properties of Se-NPs dyed wool fabrics at different temperatures.

Se-NPs conc.	Temp. °C	UV Protection Properties		
Blank wool		UV-A Transmittance	UV-B Transmittance	UPF
		10.43	1.72	33.46
12.5 mM	At room temp.	2.33	1.58	62.5 (60.2) ^1^
25 mM		1.51	1.37	81.8 (80)
37.5 mM		1.46	0.83	120 (117)
50 mM		1.21	0.36	255 (250)
12.5 mM	At 40 °C	1.44	1.23	90 (87)
25 mM		1.32	0.96	130 (125.6)
37.5 mM		1.04	0.54	189.5 (180.7)
50 mM		0.76	0.21	274 (270)
12.5 mM	At 70 °C	0.71	0.88	133.5 (130.2)
25 mM		0.52	0.73	176 (170.5)
37.5 mM		0.35	0.61	258.7 (255)
50 mM		0.27	0.42	321 (320)
12.5 mM	At 100 °C	0.59	0.77	170.2 (166)
25 mM		0.5	0.65	210 (204.8)
37.5 mM		0.41	0.32	266.9 (260)
50 mM		0.08	0.06	452.6 (447.5)

^1^ Values in parentheses indicate durability of UV protection properties after 10 washing cycles.

**Table 3:** Color characteristics for Se-dyed wool fabrics at different temperatures.

Se-NPs Conc.	Temp. °C	L *	a *	b *	c *	H *	K/S
Blank wool	Room temp.	86.87	0.96	12.33	12.37	85.56	1.1
12.5 mM		79.77	7.83	20.2	21.67	68.81	2.21 (2)^1^
25 mM		67.81	18.84	26.05	32.15	60.12	3.71 (3.5)
37.5 mM		68.77	17.97	28.98	34.1	58.19	4.68 (4.3)
50 mM		59.14	24.52	29.26	37.41	49.05	6.63 (6.4)
12.5 mM	40 °C	75.56	11.47	21.36	24.24	61.77	2.53 (2.5)
25 mM		64.14	22.06	27.21	32.75	57.66	3.34 (3.2)
37.5 mM		66.9	23.85	29.28	32.15	51.86	4.23 (4)
50 mM		42.32	27.34	30.12	39.77	44.24	6.31 (6)
12.5 mM	70 °C	42.16	12.82	15.1	19.81	49.67	9.8 (9.5)
25 mM		42.76	18.03	21.51	28.07	50.02	12.7 (12.3)
37.5 mM		42.26	24.63	28.37	37.57	49.04	15.3 (15)
50 mM		36.03	34.3	27.04	42.25	49.99	16.3 (16)
12.5 mM	100 °C	33.21	5.76	9.09	10.76	57.63	13.8 (13.3)
25 mM		29.27	13.56	13.56	18.44	42.63	18.2 (18)
37.5 mM		28.42	16.96	16.96	23.01	42.53	21.7 (21.4)
50 mM		28.43	17.06	17.06	23.15	42.53	22.1 (22)

* Values in parentheses indicate color strength (K/S) properties after 10 washing cycles.

**Table 4:** Fastness properties of Se-dyed wool fabrics at different temperatures.

Se-NPs Conc.	Temp. °C	WF ^1^		RF ^2^		LF ^3^
		Alt.	St.	Wet	Dry	
12.5 mM	Room temp.	5 (5) ^4^	5 (5)	5 (5)	5 (5)	4/5 (4/5)
25 mM		5 (5)	5 (5)	5 (5)	5 (5)	4/5 (4/5)
37.5 mM		5 (5)	5 (5)	5 (5)	5 (5)	4/5 (4/5)
50 mM		5 (5)	5 (5)	5 (5)	5 (5)	4/5 (4/5)
12.5 mM	40 °C	5 (5)	5 (5)	5 (5)	5 (5)	4/5 (4/5)
25 mM		5 (5)	5 (5)	5 (5)	5 (5)	4/5 (4/5)
37.5 mM		5 (5)	5 (5)	5 (5)	5 (5)	4/5 (4/5)
50 mM		5 (5)	5 (5)	5 (5)	5 (5)	4/5 (4/5)
12.5 mM	70 °C	4/5 (4/5)	4/5 (4/5)	4/5 (4)	4/5 (4/5)	4 (4)
25 mM		4/5 (4/5)	4/5 (4/5)	4 (4)	4/5 (4/5)	4 (4)
37.5 mM		4/5 (4/5)	4/5 (4/5)	4 (4)	4/5 (4/5)	4 (4)
50 mM		4/5 (4/5)	4/5 (4/5)	4 (4)	4/5 (4/5)	4 (4)
12.5 mM	100 °C	4/5 (4)	4/5 (4)	3/4 (3)	4 (4)	4 (4)
25 mM		4/5 (4)	4/5 (4)	3/4 (3)	4 (4)	4 (4)
37.5 mM		4/5 (4)	4/5 (4)	3/4 (3)	4 (4)	4 (4)
50 mM		4/5 (4)	4/5 (4)	3/4 (3)	4 (4)	4 (4)

^1^ wash fastness. ^2^ rubbing fastness. ^3^ light fastness. ^4^ Values in parentheses indicate durability of fastness properties after 10 washing cycles.

**Table 5:** Mechanical properties of blank and Se-dyed wool fabrics at different temperatures.

Samples	Mechanical Properties	
	Tensile Strength (N/cm^2^)	Elongation (%)
Blank wool	356.8	26.95
Se-dyed wool at RT.	330.8	25.54
40 °C	314.5	23.22
70 °C	284.4	20.22
100 °C	276	19.94
120 °C	176.6	13.88

## Data Availability

The data presented in this study are available on request from the corresponding author.
